# Transcriptomic study of anastasis for reversal of ethanol-induced apoptosis in mouse primary liver cells

**DOI:** 10.1038/s41597-022-01470-8

**Published:** 2022-07-18

**Authors:** Ho Man Tang, C. Conover Talbot, Ming Chiu Fung, Ho Lam Tang

**Affiliations:** 1 School of Life Sciences, Chinese University of Hong Kong, Shatin, Hong Kong SAR China; 2 Institute for Basic Biomedical Sciences, Johns Hopkins University School of Medicine, Baltimore, MD 21205 USA; 3 Department of Neurosurgery, Johns Hopkins University School of Medicine, Baltimore, MD 21205 USA

**Keywords:** Apoptosis, Transcriptomics

## Abstract

Anastasis is a cell recovery mechanism that rescues dying cells from the brink of death. Reversal of apoptosis is the first example of anastasis. Here, we describe a comprehensive dataset containing time-course mRNA expression profiles for reversal of ethanol-induced apoptosis in mouse primary liver cells *in νitro*. This transcriptome dataset includes the conditions of the untreated cells, cells undergoing apoptosis triggered by incubating with cell death inducer of 4.5% ethanol for 5 hours, and apoptosis reversal of ethanol-induced cells at the early (3^rd^ hour), middle (6^th^ hour), and late (24^th^, 48^th^ hour) stages after being washed with and incubated in fresh cell culture medium. By comparing this dataset with the transcriptomic profiles of other anastasis models generated with different combinations of cell types and cell death inducers, investigators can identify the key regulators governing reversal of apoptosis and other reversible cell death processes. Therefore, reusing or reanalysing this dataset will facilitate the future studies on the physiological, pathological, and therapeutic implications of anastasis.

## Abstract (summary)

**Table:** 

Measurement(s)	Microarray Analysis
Technology Type(s)	Microarray Analysis
Sample Characteristic - Organism	Mus musculus

## Background & Summary

Programmed cell death such as apoptosis has long been assumed as a one-way process, ending in cell demise^1–5^. Challenging this prevailing view, we have discovered that this cell suicide process can be reversible in primary cells and cell lines, even in dying cells that have experienced critical apoptotic events^6–13^. These events include mitochondrial outer membrane permeabilization (MOMP) for cytochrome *c* release, activation of execution caspase proteases for cleaving cellular substrates, DNA damage caused by apoptotic DNases, exposure of “eat me” signal of phosphatidylserine to cell surface, cell shrinkage and plasma membrane blebbing due to the destruction of cellular structure components, and formation of apoptotic bodies that dying cells fragment into small pieces for engulfment by phagocytes or neighbouring cells^6–13^. By developing and using a novel biosensor namely CaspaseTracker, we further discovered that reversal of apoptosis can occur at egg chambers in fruit flies after transient exposure to apoptosis-inducing environmental insults, such as protein starvation and cold shock^8,13^. We coined a term “*anastasis*” (Αναστάσης), a Greek word that means “rising to life”, to describe the recovery of dying cells^7,13^. Our discovery of anastasis *in vitro* and *in vivo* has been corroborated independently in subsequent studies^14–19^, indicating that anastasis is a general phenomenon.

Anastasis has important physiological, pathological, and therapeutic implications^13^. Anastasis could be an unrecognized cell survival mechanism to protect cells that are difficult to replace, such as cardiomyocytes and neurons^13,20^. If this is true, enhancing anastasis to avert apoptosis and help spare injured heart cells and neurons might be beneficial for treating heart failure and brain injury, respectively. Anastasis could also be an unexpected escape mechanism used by cancer cells to survive cell death-inducing cancer therapy, causing cancer recurrence^13^. Therefore, suppressing anastasis in dying cancer cells could be a novel therapeutic strategy to cure cancers by inhibiting cancer recurrence^13^. Furthermore, anastasis could be a normal physiological mechanism that mediates the decision of cell death and survival, such as for embryonic development and normal homeostasis in multicellular organisms^13^. A better understanding of anastasis regulation is critical to leverage this cell recovery process for therapeutic applications by controlling cell death and survival.

**Fig. 1:**
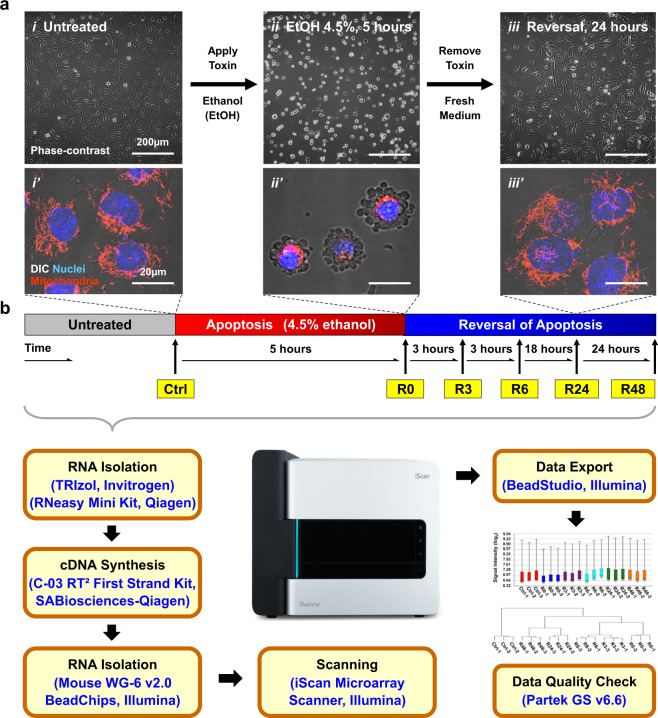
Overview of experimental design and study workflow. **(a)** Reversal of ethanol-induced apoptosis in mouse primary liver cells *in νitro* served as a model for this microarray study. Phase-contrast images of (*i*) untreated cells, (*ii*) cells undergoing apoptosis triggered by 4.5% ethanol (EtOH) for 5 hours, and (***iii***) ethanol-treated cells washed with and then incubated in fresh medium for 24 hours. (***i’-iii’***) Merged confocal images, with conditions like *i-iii*, for visualizing mitochondria (red), nuclei (blue), and cell morphology (DIC). **(b)** An outline of the workflow for the time-course microarray study. Mouse primary liver cells were treated with 4.5% ethanol for 5 hours to induce apoptosis (R0), and then were washed with and further incubated in fresh culture medium for 3 hours (R3), 6 hours (R6), 24 hours (R24), and 48 hours (R48) to allow reversal of apoptosis. Untreated cells served as control (Ctrl). Cell samples of three biological replicates from each condition mentioned above were collected for RNA extraction, analysed using Illumina Mouse WG-6 v2.0 Expression BeadChip for whole-genome expression profiling, and performed quality check by Partek Genomics Suite (GS) v6.6.

To harness anastasis for developing innovative therapies, a critical step is to identify the anastasis regulators, as these are potential druggable therapeutic targets. Yet, the molecular mechanisms governing anastasis are unclear. Therefore, we performed the first time-course whole-genome gene expression study to elucidate the molecular signature of anastasis using Illumina BeadChip microarrays (Fig. 1)^7,10^. We selected the reversal of ethanol-induced apoptosis in mouse primary liver cells *in vitro* as the first model to study anastasis, because ethanol is a potent apoptosis inducer to the liver cells^21^, and uniform and robust reversal response in apoptotic dying liver cells after removal of ethanol has been observed in our studies (Fig. 1ai-iii, ai’-iii’)^7,10^.

Healthy mouse primary liver cells displayed a flat spreading morphology and tubular mitochondria extended throughout the cytosol (Fig. 1ai-i’). To trigger apoptosis, the liver cells were incubated with cell death inducer 4.5% ethanol in cell culture medium for 5 hours. When a majority of the cells displayed hallmarks of apoptosis, such as mitochondrial fragmentation, nuclear condensation, plasma membrane blebbing, and cell shrinkage (Fig. 1aii-ii***’***), they were washed with and incubated in fresh culture medium for reversal of apoptosis (Fig. 1aiii-iii***’***). The molecular signature of apoptosis reversal was captured by microarray analysis at the early (3^rd^ hour), middle (6^th^ hours) and late (24^th^ and 48^th^ hours) time points after removal of ethanol. Untreated cells and apoptotic cells served as controls (Fig. 1b).

The present dataset has significant reuse value for future research to identify the key anastasis regulators. Illustrating differential gene expression is an important strategy to uncover regulators for the normal physiological or pathological process^22^. However, it could be difficult to identify the key anastasis regulators by simply comparing the changes in gene expression between anastatic cells and the controls (e.g. untreated and apoptotic cells) in a single dataset, because many of the changes could be due to the specific cell responses caused by different cell death inducers. Therefore, it is of great interest to identify changes in gene expression shared between different anastasis models, such as cell recovery from different death inducers and cell types. While the different cell death inducers could trigger cell death differently in different cell types (thereby causing their unique signatures), some “core” features of anastasis are expected to be conserved between different anastasis models (corresponding to apoptosis, an evolutionarily conserved process^23^). Therefore, future investigations comparing the present dataset with others can facilitate studies for identifying the regulatory mechanisms and the nature of anastasis.

## Methods

### Cell culture

Mouse primary liver cells were isolated from 4 to 6-weeks-old BALB/c mice by enzymatic digestion of collagenase according to the manufacturer’s protocols (Worthington Biochemical Corporation, Lakewood, NJ). The isolated liver cells were pooled and cultured in DMEM/F-12 (Dulbecco’s Modified Eagle Medium/Nutrient Mixture F-12) supplemented with 10% fetal bovine serum (FBS), 100 units/mL penicillin, and 100 μg/mL streptomycin (Life Technologies, Carlsbad, CA), with 80% cell confluency onto tissue culture dishes (Corning, NY) at 37 °C with 5% CO_2_/95% air as we previously described^10^. The same source of liver cells was used for all microarray experiments.

### Administration and removal of apoptosis inducer

Mouse primary liver cells were plated onto tissue culture dishes (Corning, NY) with 80% cell confluency, and were cultured in the optimal condition as described above for 24 hours before being exposure to apoptosis inducer. To induce apoptosis, the liver cells were incubated with 4.5% ethanol in cell culture medium (vol/vol) for 5 hours. To remove apoptosis inducer, the ethanol-induced dying cells were washed 3 times with fresh cell culture medium, and then cultured in the fresh medium in the optimal condition as described above for cell recovery.

### Live-cell microscopy

Live-cell imaging to capture apoptosis and reversal of apoptosis was performed as we described^10^. In brief, liver cells were incubated 20 minutes with 50 nM Mitotracker Red CMXRos for staining mitochondria, and 250 ng/mL Hoechst 33342 for staining nuclei in culture medium (Invitrogen, Carlsbad, CA). The cells were washed 3 times, and then cultured in fresh culture medium for 10 minutes before subjecting to microscopy. Cell images were captured using Zeiss LSM 800 inverted confocal microscope with a 10x, NA 0.3 Plan-Neofluar objective, or a 63x NA 1.3 Plan-Apochromat objective. Cell morphology was imaged by differential interference contrast (DIC) microscopy. Cell images were analysed using Zen and AxioVision software (Carl Zeiss, Jena, Germany).

### Cell sample collection

Three biological replicates were performed in the conditions of untreated cells (Control, Ctrl), apoptotic dying cells treated with 4.5% ethanol for 5 hours (R0), and the treated cells that were washed with and further incubated in the fresh culture medium to reverse apoptosis for 3 hours (R3), 6 hours (R6), 24 hours (R24), and 48 hours (R48) as we previously described (Fig. 1)^10^.

### RNA collection, isolation, and quality check

To harvest the total RNA from the corresponding cell conditions, the cells were washed once with phosphate-buffered saline (PBS) to remove the cell culture medium, and then were immediately lysed by mixing thoroughly with TRIzol reagent (Life Technologies) using pipetting with 1 × 10^7^ cells/mL on the cell culture dish. The total RNA was purified using RNeasy Mini Kit according to the manufacturer’s protocols (QIAGEN, Cologne, Germany).

The isolated RNA was quantified by NanoDrop ND-1000 spectrophotometer (NanoDrop Technologies, Wilmington, DE). The intactness of RNA was determined by using Eukaryote Total RNA Nano assay on the Agilent 2100 Bioanalyzer system, and evaluated by RNA integrity number (RIN) software algorithm according to the manufacturer’s instructions^24^ (Agilent technologies, Santa Clara, CA).

### cDNA construction, microarray hybridization, and data acquisition

Generation of complementary DNA (cDNA) and microarray hybridization were conducted at the SABiosciences Core Facility using the standard protocols from Illumina (San Diego, CA) and QIAGEN. In brief, isolated RNA was used to construct cDNA labelling with a fluorescent dye Cy3 by using SABiosciences C-03 RT^2^ First Strand Kit (SABiosciences-QIAGEN, Frederick, MD) as described^25^. The cDNA was hybridized to Illumina Mouse WG-6 v2.0 Expression BeadChip arrays, which consist of 45,281 probe for each sample. Arrays were scanned with the Illumina iScan microarray scanner.

### Data extraction and analysis

Data from the Illumina iScan was extracted by Illumina BeadStudio software using the Gene Expression module to generate foreground signal intensity values, subtract background signals, and perform data normalization with the default protocol (Illumina, San Diego, CA)^26^. Processed Illumina signal values were imported into the Partek Genomics Suite v6.6 (Partek, St. Louis, MO, USA)^27^ platform where signal values were converted into log_2_ space for quality control and to evaluate gene expression levels. For quality control, the raw log_2_ values of all genes underwent box plot analysis, and hierarchical clustering to create a clustering dendrogram.

## Data Records

The dataset for reversal of ethanol-induced apoptosis in mouse primary liver cells was deposited to the Gene Expression Omnibus (GEO) database with the accession number of GSE90959^28^, and is available for public access.

The data can be accessed through this link: https://identifiers.org/geo:GSE90959

The GEO accession numbers for individual samples are listed in Table 1.

**Table 1:** Gene Expression Omnibus (GEO) accession number of individual samples in the transcriptome dataset for reversal of ethanol-inducted apoptosis in mouse primary liver cells^10^.

Sample	Sample Description	GEO Accession	Ref.
Ctrl-1	Ctrl (Untreated mouse primary liver cells) Replicate 1	GSM2418377	^43^
Ctrl-2	Ctrl (Untreated mouse primary liver cells) Replicate 2	GSM2418378	^44^
Ctrl-3	Ctrl (Untreated mouse primary liver cells) Replicate 3	GSM2418379	^45^
R0-1	R0 (Liver cells were treated with 4.5% ethanol in culture medium for 5 hours) Replicate 1	GSM2418380	^46^
R0-2	R0 (Liver cells were treated with 4.5% ethanol in culture medium for 5 hours) Replicate 2	GSM2418381	^47^
R0-3	R0 (Liver cells were treated with 4.5% ethanol in culture medium for 5 hours) Replicate 3	GSM2418382	^48^
R3-1	R3 (Treated cells were washed with fresh culture medium, and then incubated for 3 hours) Replicate 1	GSM2418383	^49^
R3-2	R3 (Treated cells were washed with fresh culture medium, and then incubated for 3 hours) Replicate 2	GSM2418384	^50^
R3-3	R3 (Treated cells were washed with fresh culture medium, and then incubated for 3 hours) Replicate 3	GSM2418385	^51^
R6-1	R6 (Treated cells were washed with fresh culture medium, and then incubated for 6 hours) Replicate 1	GSM2418386	^52^
R6-2	R6 (Treated cells were washed with fresh culture medium, and then incubated for 6 hours) Replicate 2	GSM2418387	^53^
R6-3	R6 (Treated cells were washed with fresh culture medium, and then incubated for 6 hours) Replicate 3	GSM2418388	^54^
R24-1	R24 (Treated cells were washed with fresh culture medium, and then incubated for 24 hours) Replicate 1	GSM2418389	^55^
R24-2	R24 (Treated cells were washed with fresh culture medium, and then incubated for 24 hours) Replicate 2	GSM2418390	^56^
R24-3	R24 (Treated cells were washed with fresh culture medium, and then incubated for 24 hours) Replicate 3	GSM2418391	^57^
R48-1	R48 (Treated cells were washed with fresh culture medium, and then incubated for 48 hours) Replicate 1	GSM2418392	^58^
R48-2	R48 (Treated cells were washed with fresh culture medium, and then incubated for 48 hours) Replicate 2	GSM2418393	^59^
R48-3	R48 (Treated cells were washed with fresh culture medium, and then incubated for 48 hours) Replicate 3	GSM2418394	^60^

GEO accession number of the dataset is GSE90959^28^ with 18 samples^43–60^.

## Technical Validation

### Validation of apoptosis and reversal of apoptosis

To harvest cell samples for studying reversal of apoptosis, it is important to ensure that apoptosis is triggered in response to cell death inducer, and reversal of apoptosis occurs after removal of the inducer.

To validate that apoptosis was triggered by the cell death inducer of 4.5% ethanol for 5 hours (R0), we performed live-cell microscopy to confirm that the ethanol-induced mouse primary liver cells displayed morphological hallmarks of apoptosis, including mitochondrial fragmentation, nuclear condensation, cell shrinkage, and plasma membrane blebbing (Fig. 1ai-ii, ai’-ii’)^10^. Plasma membrane blebbing is the consequence of caspase-3 activation^29^, which is the biochemical hallmark of apoptosis^2^, thereby indicating the presence of active effector caspase activity in the ethanol-induced apoptotic dying liver cells. Our earlier studies further demonstrated the cleavage (activation) of caspase-3 and its substrate PARP to occur in the ethanol-treated liver cells by using Western blot analysis under the same condition^7,10^. Taken together, these studies evidence that the mouse primary liver cells underwent apoptosis in response to the ethanol induction.

To validate that reversal of apoptosis occurred after removal of ethanol, we performed live-cell microscopy to confirm that the morphological hallmarks of apoptosis vanished after the ethanol-induced apoptotic dying cells had been washed and incubated with fresh medium for 24 hours, and the recovered cells regained normal cell morphology (Fig. 1aiii,aiii***’***)^10^. Our Western blot analysis in our earlier studies also demonstrated reversal of apoptosis that the cleaved caspase-3 and PARP vanished at 24 hours after removal of ethanol^7,10^. The disappearance of apoptosis hallmarks was due to reversal of apoptosis, rather than the dead cells were washed away. It is because we performed a time-course live-cell microscopy to monitor the same group of liver cells throughout the experiment, and confirmed that the condensed and blebbing dying cells stayed on the surface of cell culture dish after being washed and then regained normal morphology^7,10^.

Under the conditions used in this study, the responses of the liver cells were remarkably uniform, undergoing apoptosis during ethanol induction and its reversal after removal of ethanol (Fig. 1aii-iii,aii’-iii***’***). These uniform liver cell responses suggest that these cells underwent highly synchronized cellular events in the given conditions, which is important for revealing their gene transcription profile that represents the general cell population.

These validations indicate that our chosen study model, reversal of ethanol-induced apoptosis in mouse primary liver cells, is suitable for characterizing the molecular signature of apoptosis reversal.

### Evaluation of RNA integrity

**Fig. 2:**
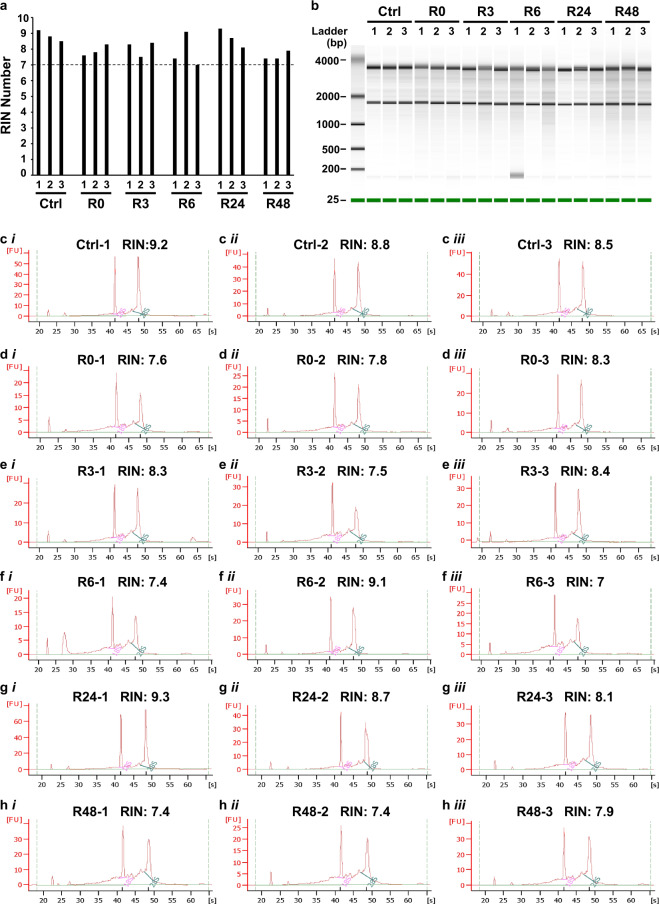
RNA integrity measurement. **(a)** A summary of RNA integrity numbers (RINs), **(b)** gel-like images, and **(c–h)** electropherograms generated by the Agilent 2100 Bioanalyzer system for determining the quality of the eighteen RNA samples (six conditions as listed in Fig. 1, three replicates for each condition). The six conditions are the untreated liver cells (ctrl), apoptotic liver cells induced with 4.5% ethanol for 5 hours (R0), and anastatic liver cells after removal of ethanol for 3 hours (R3), 6 hours (R6), 24 hours (R24), and 48 hours (R48). The 18 S and 28 S ribosomal bands are marked on the electropherograms. Fluorescence unit: FU; Time in second: S.

Acquiring high quality RNA for microarray analysis is important for accuracy of gene expression study. The quality of total RNA of all samples was measured by Agilent 2100 Bioanalyzer system using Eukaryote Total RNA Nano assay, and was evaluated by RNA integrity number (RIN) software algorithm for assigning integrity values to RNA measurement, with range from 10 (intact) to 1 (totally degraded)^24^. The RIN score of all the RNA samples in this study was 7 or above (Fig. 2a). The gel-like images (Fig. 2b) and electropherograms (Fig. 2c–h) generated by the Bioanalyzer revealed the peak signal of the 18 S and 28 S ribosomal bands for each sample, indicating that the RNA collected from all samples had high quality and was suitable for microarray analysis.

### Data distribution and quality check

For microarray analysis, quality controls were performed to evaluate the range of signal intensity level of all eighteen samples, and to determine the similarity of transcriptional profiles between biological replicates among all the samples (6 conditions, 3 replicates for each condition, covering Ctrl, R0, R3, R6, R24, and R48).

**Fig. 3:**
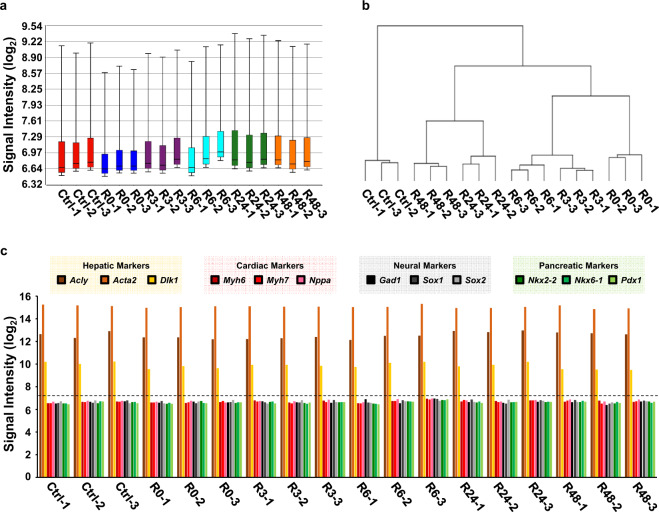
Quality check dataset. **(a)** Signal box plot illustrating the raw data distribution of eighteen samples in the dataset, depicting the log_2_ value of probe signal intensity of median, interquartile range, and whiskers of each sample. **(b)** Dendrogram of all eighteen samples’ raw log_2_ signals generated using hierarchical clustering. **(c)** Signal intensity of hepatic marker genes (*Acly*, *Acta2*, *Dlk1*), cardiac marker genes (*Myh6*, *Myh7*, *Nppa*), neural marker genes (*Gad1*, *Sox1*, and *Sox2*), and pancreatic marker genes (*Nkx2-2*, *Nkx6-1*, *Pdx1*) of eighteen samples in the dataset. Dotted line indicates the overall average signal in the dataset (log_2_ 7.3).

First, overall data distribution of all the samples was summarized in a signal box plot, which reveals the raw log_2_ signal intensity level of each sample (Fig. 3a), with the interquartile range (IQR) to cover the lower quartile (Q1, 25%) to the upper quartile (Q3, 75%) of the signal range, and with whiskers to cover the variability of the signal outside the lower and upper quartiles (10% to 90%). The signal box blot demonstrates uniform distribution of signal intensity in all the samples.

Second, the similarity of biological replicates was evaluated by hierarchical clustering, using Pearson’s Dissimilarity and average linkage, which compared the gene expression profiles between all eighteen samples (6 conditions, 3 replicates as mentioned above), and grouped them based on their similarity in the transcription profile within this dataset. Our hierarchical dendrogram illustrates that all the biological replicates in the same time point are grouped and connected with each other with the shortest branches (Fig. 3b). This dendrogram reveals that all the biological replicates share the highest similarity in their gene expression pattern within their corresponding time points. This also indicates the distinct difference of the gene expression pattern between different time points of cells that reverse apoptosis (R3, R6, R24, R48), as well as the untreated cells (Ctrl) and apoptotic (R0) cells.

### Expression of hepatic markers

All eighteen samples in the dataset were evaluated for the expression of hepatic marker genes (Fig. 3c). The raw signal of the hepatic marker genes such as *Acly*^30^, *Acta2*^31^, and *Dlk1*^32^ (log_2_ signal >9.48) is higher than the overall average signal intensity (7.30) in the dataset (Fig. 3c). It is also higher than the signal (<7.00) of cardiac marker genes (*Myh6*/*alphaMHC*, *Myh7*/*betaMHC*, *Nppa*)^33,34^, neural marker genes (*Gad1*/*GAD67*, *Sox1*, and *Sox2*)^35–37^, and pancreatic marker genes (*Nkx2-2*, *Nkx6-1*, *Pdx1*)^38^ in all the samples (Fig. 3c). The expression of hepatic marker genes suggests the presence of mouse primary liver cells in all the samples that were collected to generate the dataset.

## Usage Notes

We used reversal of apoptosis as the first example of anastasis^6,7,10,13^, because apoptosis is the most well-studied form of programmed cell death. To date, there are two datasets about the time-course gene expression profiles of apoptosis reversal published. They are reversal of ethanol-induced apoptosis in mouse primary liver cells^10^, and reversal of ethanol-induced apoptosis in human cervical carcinoma HeLa cells^16^. Identifying the molecular signatures shared between these datasets could reveal the candidates of anastasis regulator. Future studies can further apply bioinformatics, such as Connective Map (CMAP)^39^ and Ingenuity Pathway Analysis (IPA)^40^ to identify the small molecules that target the anastasis regulators. These agents will be the useful tools for understanding the consequences of anastasis, and for exploring the therapeutic potentials of manipulating anastasis to improve health outcomes. Moreover, we and others have suggested that different cell death processes, such as ferroptosis and necroptosis, are potentially reversible, thereby adding emerging members of anastasis^13^. By illustrating and comparing the gene expression profiles of different reversible cell death processes, future investigations can elucidate the molecular mechanisms and druggable regulators for mediating cell recovery. These studies will provide important insights into the physiological functions, pathological consequences, and therapeutic implications of anastasis.

## Data Availability

All parameters required for using Illumina BeadStudio software to generate summary probe profile file of the present dataset are publicly available in “The BeadStudio Gene Expression Module v3.4 User Guide” from Illumina^26^. It includes information about the data that can be exported from the “Group”, “Sample”, and “Control” Probe Profile tables. The probe profile tables include the average signal intensities (AVG_Signal) detected from all the beads of a given beadtype which target a specific sequence in the mouse genome. The ‘Detection Pval’ is the p-value that represents the chance that the target sequence signal was distinguishable from negative controls. Data analysis in this manuscript was performed by using Agilent 2100 Bioanalyzer^24^ and Partek Genomics Suite v6.6^27^ software, and their default protocols. Input files generated by BeadStudio and used for data analysis^41^ and tables with analysis output data^42^ are available at *Figshare*.
